# Extracellular Vesicles as Biological Templates for Next-Generation Drug-Coated Cardiovascular Devices: Cellular Mechanisms of Vascular Healing, Inflammation, and Restenosis

**DOI:** 10.3390/cells15020121

**Published:** 2026-01-09

**Authors:** Rasit Dinc, Nurittin Ardic

**Affiliations:** 1 INVAMED Medical Innovation Institute, New York, NY 10007, USA; 2 Med-International UK Health Agency Ltd., Nuneaton CV11 6LT, Warwickshire, UK; nurittinardic@yahoo.com

**Keywords:** extracellular vesicles, exosomes, drug-eluting stents, endothelial cells, smooth muscle cells, vascular healing, restenosis, inflammation, cardiovascular devices, biomimetic nanoparticles

## Abstract

**Highlights:**

**What are the main findings?**

- Extracellular vesicle (EV) biology offers a mechanistic roadmap for redesigning drug-coated cardiovascular devices beyond non-selective antiproliferative drug delivery.
- EV-inspired coating strategies can integrate pathway-information-based cargo signaling and surface interactions to coordinate endothelial healing, smooth muscle responses, and inflammation resolution.

**What is the implication of the main findings?**

- EV-inspired devices can reduce the trade-off between restenosis prevention and delayed vascular healing by delivering cell-directed drug delivery that promotes healing at the injury site.
- Implementation will require standardized EV characterization, scalable manufacturing/quality control, and clinically relevant endpoints that capture both efficacy and quality of healing.

**Abstract:**

While drug-eluting cardiovascular devices, including drug-eluting stents and drug-coated balloons, have significantly reduced restenosis rates, they remain limited by delayed vascular healing, chronic inflammation, and late adverse events. These limitations reflect a fundamental mismatch between current device pharmacology, which relies on nonselective antiproliferative drugs, and the highly coordinated, cell-specific programs that orchestrate vascular repair. Extracellular vesicles (EVs), nanometer-scale membrane-bound particles secreted by virtually all cell types, provide a biologically evolved platform for intercellular communication and cargo delivery. In the cardiovascular system, EVs regulate endothelial regeneration, smooth muscle cell phenotype, extracellular matrix remodeling, and macrophage polarization through precisely orchestrated combinations of miRNA, proteins, and lipids. Here, we synthesize mechanistic insights into EV biogenesis, cargo selection, recruitment, and functional effects in vascular healing and inflammation and translate these into a formal framework for EV-inspired device engineering. We discuss how EV-based or EV-mimetic coatings can be designed to sense the local microenvironment, deliver encoded biological “instruction sets,” and function within ECM-mimetic scaffolds to couple local stent healing with systemic tissue repair. Finally, we outline the manufacturing, regulatory, and clinical trial issues that must be addressed for EV-inspired cardiovascular devices to transition from proof of concept to clinical reality. By shifting the focus from pharmacological suppression to biological regulation of healing, EV-based strategies offer a path to resolve the long-standing tradeoff between restenosis prevention and durable vascular healing.

## 1. Introduction

Despite significant advances in revascularization and device-based therapies, cardiovascular disease remains the leading cause of death globally. While drug-eluting stents (DES) and DCB have significantly reduced the risk of restenosis, they continue to face persistent biological limitations: delayed endothelial healing, chronic inflammation, impaired vasomotion, and a non-negligible rate of late thrombosis [1,2,3]. These shortcomings reflect a deeper conceptual problem: current drug-coated devices rely on non-selective cytostatic agents that indiscriminately suppress cell proliferation rather than coordinating the multicellular processes required for durable vascular repair. Consequently, device pharmacology remains mismatched with the biological complexity of the injured vessel wall, and the global burden of cardiovascular disease continues to increase despite technical advances in percutaneous coronary intervention [4,5].

Extracellular vesicles (EVs) offer an alternative design paradigm based on endogenous repair mechanisms. EVs deliver spatially and temporally controlled “instruction sets” to endothelial cells, SMCs, macrophages, and fibroblasts, orchestrating intercellular communication through precisely packaged microRNAs, proteins, lipids, and metabolites [6,7,8]. Their biogenesis integrates ESCRT-dependent and lipid-driven pathways; cargo selection is governed by RNA-binding protein-motif interactions; and their uptake involves clathrin- and caveolin-mediated endocytosis, macropinocytosis, and direct membrane fusion [9,10,11]. Through these mechanisms, EVs not only regulate isolated pathways but also coordinate vascular regeneration, restore endothelial integrity, regulate smooth muscle cell phenotypes, resolve inflammation, and remodel extracellular matrix architecture [12,13,14]. These mechanistic insights reveal a profound mismatch between endogenous repair biology and current device technologies. While DES and DCBs release small molecules that broadly and passively inhibit proliferation, EVs sense microenvironmental cues, transmit encoded biological signals, and move within a structural matrix that shapes their dissemination, uptake, and functional reach [15,16]. Therefore, EV-inspired coatings can incorporate microenvironment-responsive release mechanisms, deliver phenotype-specific miRNA and protein cargo, interface with ECM-mimetic scaffolds to regulate cell behavior, and uniquely combine local vascular healing with systemic regenerative effects [17,18,19]. Emerging proof-of-concept studies, including exosome-eluting stents, enzyme-responsive vesicle delivery systems, and cell-free regenerative constructs, demonstrate the feasibility and transformative potential of this approach [17,20,21].

In this review, we synthesize current mechanistic knowledge of EV biology and integrate it into a formal framework for EV-inspired cardiovascular device engineering. We analyze the molecular and cellular pathways by which EVs regulate vascular repair, examine preclinical evidence supporting their therapeutic benefits, and summarize engineering strategies that translate EV principles into next-generation devices [8,22,23]. Finally, we discuss the manufacturing, regulatory, and clinical considerations required for the translation of EV-based or EV-mimetic technologies to human cardiovascular intervention [24,25,26,27]. By reframing device pharmacology through the lens of endogenous vesicle signaling, we identify a pathway to resolve the long-standing tradeoff between restenosis prevention and durable vascular healing [12,19,28].

A graphical summary is provided to visually outline the core concept of cardiovascular device design inspired by electric vehicles.

## 2. Extracellular Vesicles: Biogenesis, Classification, and Biological Functions

### 2.1. EV Classification and Nomenclature

EVs are defined by Minimum Information for Extracellular Vesicle Studies 2023 [29] as lipid bilayer-delimited particles released from cells do not replicate on their own. MISEV2023 proposes operational classification primarily based on measurable features; this classification typically includes small EVs (sEVs, <200 nm, predominantly exosomes) and large EVs (lEVs, >200 nm), as well as other EV-associated particles such as apoptotic bodies [29]. In terms of historical context, the terms “exosomes” and “microvesicles” are briefly introduced here; for the remainder of the article, we will use the operational terms sEVs and lEVs to reflect that EV size alone does not determine biogenesis or the release pathway [29,30].

**Figure 1:**
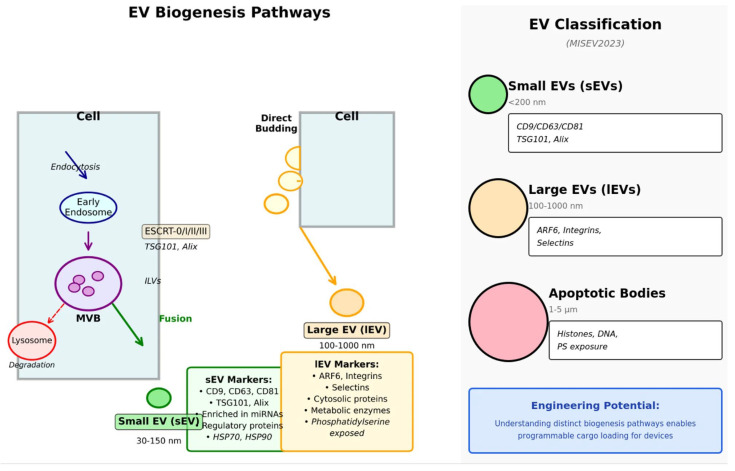
Extracellular vesicle biogenesis and classification. EV biogenesis occurs via ESCRT-dependent pathways (early endosome → MVB → small EV release) or direct plasma membrane budding (large EVs). The key mechanism involves ESCRT complex components (TSG101, Alix) for small EVs. Classification according to MISEV2023 guidelines: small EVs (<200 nm) labeled with CD9/CD63/CD81 and TSG101; large EVs (100–1000 nm) labeled with ARF6 and integrins; apoptotic bodies (1–5 μm) containing histones and DNA. Understanding different biogenesis pathways enables the engineering of EV mimicry platforms with programmable cargo loading. ESCRT, endosomal sorting complex required for transport; EV, extracellular vesicle; MISEV, Minimum Information for Extracellular Vesicle Studies; MVB, multiple vesicular bodies.

From a biogenesis perspective, many sEVs arise via inward budding of endosomal membranes and the formation of intraluminal vesicles (ILVs) within multiple vesicular bodies (MVBs). This process may involve ESCRT-dependent cargo sorting and membrane cleavage, as well as ESCRT-independent lipid-driven mechanisms [30,31,32]. Once formed, MVBs can associate with the plasma membrane, releasing ILVs as sEVs, or they may instead be directed to lysosomes for degradation [30,31,33]. lEVs are generally directly associated with plasma membrane budding, highlighting that EV heterogeneity reflects both intracellular transport pathways and the activation state of the host cell [29,32] (Figure 1).

Large EV(s) (ectosomes) with a diameter of 100–1000 nm are produced by direct outward budding from the plasma membrane [6,30]. Apoptotic bodies (1–5 μm) are released during programmed cell death and contain cellular debris and organelles [34]. While these biogenesis-based terms remain conceptually useful, MISEV2023 highlights that precise characterization of subtypes remains technically challenging [29]. The classification and key features of the three main EV categories are summarized in Table 1, which provides a comprehensive overview of their biogenesis pathways, molecular markers, cargo composition, and primary biological functions.

### 2.2. Molecular Cargo and Biological Functions

EVs carry a structured repertoire of proteins, lipids, and nucleic acids that reflect cellular state and environmental cues, enabling functional communication between cardiovascular cell types [6,7,22]. Importantly, the EV content is not a random sampling of the cytoplasm; it is shaped by selective and stimulus-sensitive sorting pathways that influence the biological effect of a particular EV population [6,22].

Among nucleic acids, miRNAs are leading mediators of EV function in cardiovascular biology and have far-reaching effects on endothelial integrity, smooth muscle phenotype, inflammation, extracellular matrix remodeling, and cardiomyocyte survival [11,35,36]. Selective enrichment of specific miRNAs and other RNAs can involve RNA-binding proteins and defined sequence motifs, linking cellular stimuli to exported “instruction sets” [6,11,14,36,37]. EV protein and lipid composition further contributes to target recognition, recruitment, and signaling [8,29,36]. This selectivity is critical for device engineering, demonstrating that the therapeutic effect depends more on the combination of defined cargo and surface features presented to the damaged vessel wall than on the vesicles themselves [6,22].

### 2.3. EV Uptake Mechanisms and Intracellular Trafficking

EV biological activity ultimately depends on interaction with recipient cells and intracellular transport after uptake. EV internalization occurs via multiple pathways, including endocytosis, phagocytosis/efferocytosis, and in some cases, direct membrane fusion; the dominant pathway depends on EV size, membrane composition, receptor–ligand coupling, and microenvironmental context [6,10,30]. In vascular cells, uptake may involve clathrin or caveolin-related processes, while larger particles are often cleared by phagocytosis mechanisms at sites of inflammation or injury [38,39,40,41].

A significant limitation regarding translation is that functional delivery is often inefficient: a substantial portion of internalized EVs enter and are degraded in endolysosomal compartments, with only a small fraction escaping to achieve cytoplasmic delivery [10]. This bottleneck is important for EV-inspired device design because surface chemistry, ligand display, and release kinetics can be optimized to support efficient uptake pathways and/or promote timely endosomal escape, thereby increasing potency for a given delivered dose [10,30].

### 2.4. Functional Roles in Cardiovascular Homeostasis and Disease

EVs contribute to cardiovascular homeostasis by coordinating intercellular signaling related to angiogenesis, vascular tone regulation, inflammatory responses, and tissue remodeling [23,37,41]. However, the effects of EVs are context-dependent: EVs released under pathological conditions may propagate inflammatory, fibrotic, and prothrombotic signaling, while EVs derived from reparative sources may promote dissolution and regeneration [22,41]. For example, platelet-derived EVs can promote coagulation through tissue factor expression and phosphatidylserine exposure [41,42].

In contrast, EVs derived from mesenchymal stem cells, cardiac progenitor cells, and cardiomyocytes have been shown to be associated with cardioprotective effects such as reduced inflammation, increased angiogenesis, and modulation of post-injury apoptosis and fibrosis [5,38,43]. Collectively, these observations motivate EV-inspired cardiovascular devices: understanding biogenesis, cargo selection, and delivery constraints provides a mechanistic basis for designing coatings that deliver defined therapeutic signals and support physiological healing responses [6,10].

## 3. Cellular Mechanisms of Vascular Healing After Device Implantation

### 3.1. The Vascular Injury Response: A Complex Cascade

Stent implantation causes profound mechanical damage to the vessel wall, triggering a coordinated cascade of biological responses that determine clinical outcome. Immediately after deployment, the endothelium sloughs off, disrupting the endothelial glycocalyx and exposing subendothelial matrix components. This leads to platelet activation, fibrin deposition, and activation of the coagulation cascade [44,45]. Three interconnected processes then shape the healing response: acute inflammation, neointimal hyperplasia triggered by SMC proliferation and migration, and re-endothelialization [28,46].

The endothelial glycocalyx (a membrane-bound layer composed of glycosaminoglycans, proteoglycans (syndecans, glypicans), and glycoproteins) forms a critical interface between blood and the endothelium. It contributes to the regulation of vascular permeability, mechanosensing, shear stress-mediated nitric oxide production, modulation of blood cell interactions, and control of vascular tone [47]. During stent deployment, this structure is mechanically denatured, glycosaminoglycan chains are severed, and soluble heparan sulfate, hyaluronan, and chondroitin sulfate are released into the circulation. Loss of the glycocalyx increases vascular permeability, promotes leukocyte and platelet adhesion, facilitates inflammatory cell extravasation, impairs vascular tone, and impairs antithrombotic and antioxidant defenses [48].

The healing response follows a characteristic temporal pattern. In the acute phase (first 24–48 h), platelet activation and fibrin deposition predominate, creating a temporary matrix that serves as a scaffold for cellular infiltration [49]. During the subacute phase (days 3–7), monocytes and macrophages accumulate in the vessel wall and release cytokines and growth factors that promote both repair and SMC activation. From day 7 onward, SMC proliferation and migration accelerate, reaching a peak in 2–4 weeks, while re-endothelialization proceeds more slowly through the migration of endothelial cells and endothelial progenitor cells (EPCs) from the stent margins and the circulation [50,51]. In DES, this re-endothelialization process remains incomplete for months, contributing to late thrombotic risk.

### 3.2. Reendothelialization: A Critical Determinant of Long-Term Success

The regeneration of a functional endothelial layer is the most important biomarker of long-term stent safety. An intact endothelium prevents thrombosis, regulates vascular tone, and limits pathological smooth muscle cell proliferation. However, endothelial recovery after stent implantation differs markedly from physiological regeneration in both speed and quality [52,53].

In bare-metal stents, endothelial coverage of stent struts is generally achieved within 2–4 weeks. In contrast, DES exhibits significantly delayed and incomplete endothelialization, which can persist for years and is strongly associated with late stent thrombosis [54,55]. Single-cell RNA sequencing has revealed that, even if the luminal surface appears covered, regenerating endothelium often exhibits an immature and dysfunctional transcriptomic profile, with increased expression of extracellular matrix and inflammatory genes and decreased expression of barrier proteins, antithrombotic factors (thrombomodulin, tissue plasminogen activator), and vasodilator mediators such as endothelial nitric oxide synthase [56,57,58,59].

Optical coherence tomography (OCT) can visualize the neointimal lining but cannot distinguish between SMC-rich neointima and true endothelial lining. Studies on porcine DES have shown that neointimal coverage can approach 100% by 4 weeks, but histological reendothelialization lags behind, reaching only approximately 50% at 4 weeks and approximately 75% at 12 weeks [60,61]. This discrepancy has important clinical implications, as decisions regarding the duration of dual antiplatelet therapy are often based on strut coverage assessments obtained with OCT [44,62].

Mechanistically, reendothelialization after stenting depends on both the migration of endothelial cells from adjacent healthy segments and the recruitment of circulating EPCs [59]. Following vascular injury, EPCs are mobilized from the bone marrow and home to denuded areas via adhesion molecules (VCAM-1, P-selectin) and chemokine gradients (SDF-1α). Antiproliferative drugs released from DES interfere with these processes by inhibiting endothelial cell migration and EPC differentiation, thus directly impairing vascular repair [63].

### 3.3. The Dual Nature of Smooth Muscle Cell Responses

Vascular smooth muscle cells exhibit remarkable phenotypic plasticity, switching between a contractile (differentiated) and synthetic (dedifferentiated) phenotype in response to environmental stimuli [19]. This transition is central to both physiological vascular remodeling and pathological restenosis.

In healthy vessels, SMCs maintain a quiescent and contractile phenotype characterized by high expression of markers such as α-smooth muscle actin (α-SMA), SM22α, calponin, smoothelin, and smooth muscle myosin heavy chain. These cells exhibit minimal proliferative activity and primarily regulate vascular tone [64,65]. After vascular injury, SMCs adopt a synthetic phenotype with decreased expression of contractile proteins, increased extracellular matrix production (collagens I and III, fibronectin, proteoglycans), increased proliferation and migration, and increased secretion of inflammatory mediators. This transition is mediated by platelet-derived growth factor (PDGF), transforming growth factor-β (TGF-β), angiotensin II, inflammatory cytokines (IL-1β, TNF-α), and mechanical stress [8].

Transient SMC activation is essential for vascular repair; however, persistent activation leads to excessive neointimal hyperplasia and in-stent restenosis [66]. Importantly, recent data suggest that SMC behavior influences endothelial healing. Segments with faster SMC coverage exhibit more efficient subsequent endothelialization; this suggests that appropriately regulated SMC responses may promote, rather than oppose, endothelial healing [65,67]. This challenges the traditional view that SMC proliferation and endothelial repair are purely antagonistic processes.

Current DES disrupt this delicately balanced system by nonselectively inhibiting proliferation in all cell types. While this effectively reduces neointimal hyperplasia, it also impairs endothelial regeneration, often resulting in a “frozen” healing state with persistent fibrin, incomplete endothelialization, and chronic inflammation [51,54].

### 3.4. Inflammatory Cell Responses and Chronic Inflammation

Macrophages play central and multifaceted roles in vascular healing after stent implantation and exhibit functional heterogeneity, ranging from proinflammatory (M1) to proresolution (M2) phenotypes [68]. The balance and temporal sequence of macrophage polarization significantly influence healing outcomes.

Following stent deployment, monocytes are attracted to the injured vessel wall. Chemokine gradients (MCP-1/CCL2, MCP-3/CCL7) and adhesion molecules (VCAM-1, ICAM-1) expressed in activated endothelium mediate this process. These monocytes initially differentiate into macrophages by adopting a proinflammatory M1 phenotype that secretes IL-1β, IL-6, TNF-α, and reactive oxygen species. M1 macrophages phagocytose debris, kill pathogens, and activate smooth muscle cells through growth factor release. However, sustained M1 activation promotes chronic inflammation, impairs healing, and contributes to late side effects [68,69]. Under appropriate conditions, macrophages transition to an M2 phenotype. This phenotype produces anti-inflammatory cytokines (IL-10, TGF-β). M2 macrophages also promote extracellular matrix remodeling via matrix metalloproteinases, promotes angiogenesis through VEGF secretion, and facilitates tissue repair [68,70]. The M2 phenotype is essential for inflammation resolution and productive healing. However, in DES, the transition from M1 to M2 polarization is temporally disrupted, and chronic M1 activation persists due to continued foreign-body reactions to polymer coatings and delayed fibrin clearance. Hypersensitivity reactions to stent polymers or drugs represent an additional inflammatory mechanism specific to DES. Histopathological examination of DES explants has revealed eosinophil and lymphocyte infiltration, suggesting delayed-type hypersensitivity reactions associated with incomplete healing and an increased risk of thrombosis [19,68,70]. While these findings have spurred the development of polymer-free DES and bioabsorbable polymer platforms, challenges remain in achieving optimal drug release kinetics [70].

### 3.5. Therapeutic Challenge: The Balance Between SMC Inhibition and Endothelial Healing

A key challenge facing current drug-eluting stent technology lies in the nonselective nature of antiproliferative drugs. Sirolimus, everolimus, zotarolimus (mTOR inhibitors), and paclitaxel (a microtubule stabilizer) effectively suppress smooth muscle cell proliferation but simultaneously impair endothelial cell proliferation, migration, and function [3,51]. This creates an inherent therapeutic paradox: mechanisms that prevent restenosis also delay healing and increase the risk of thrombosis. To address this paradox, several strategies have been explored, including polymer-free DES, biodegradable polymer DES, dual-drug DES combining antiproliferative and pro-endothelial agents, and surface modifications that promote selective endothelial cell adhesion [19,68]. While these approaches have yielded incremental improvements, none have fundamentally resolved the tension between inhibiting neointimal hyperplasia and promoting endothelial healing.

This biological impasse has prompted the exploration of fundamentally different approaches that exploit, rather than suppress, natural healing mechanisms. Platforms inspired by extracellular vesicles offer a conceptually different strategy: instead of using synthetic drugs to nonselectively inhibit proliferation, biomimetic nanocarriers can modulate smooth muscle cell phenotype toward contractile states rather than synthetic states, while simultaneously delivering biological signals that selectively promote endothelial regeneration [15,19]. This represents a shift from pharmacological suppression to biological reprogramming, that is, from inhibition to intelligent modulation of cellular responses.

**Figure 2:**
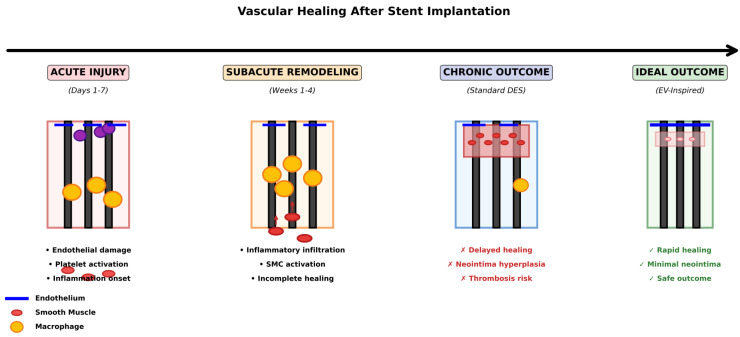
Temporal progression of vascular healing after drug-eluting stent implantation. The cartoon illustrates four phases: (Acute Injury, Days 1–7) endothelial shedding, platelet activation, and initiation of inflammation; (Subacute Remodeling, Weeks 1–4) inflammatory cell infiltration, smooth muscle cell (SMC) activation and migration, and incomplete endothelial sheath; (Chronic Outcome with Standard DES) excessive neointimal hyperplasia, incomplete re-endothelialization, chronic inflammation, and risk of late thrombosis; (Ideal Outcome with EV-Inspired Devices) rapid re-endothelialization, minimal neointima, resolved inflammation, and balanced SMC response. Cell types: endothelial cells (blue), SMCs (red), macrophages (yellow), platelets (purple). Visual Indicators: Stent wires (vertical bars, black), temporal progression (black arrow), acute injury phase (pink border), subacute remodeling phase (orange border), chronic outcome phase (light blue border), ideal outcome phase (green border). DES, drug-eluting stent; EV, extracellular vesicle; SMC, smooth muscle cell.

Figure 2 presents a temporal overview of vascular healing following stent implantation, comparing the acute inflammatory phase (0–48 h), the subacute proliferative phase (3–14 days), and the chronic remodeling phase (weeks to months). The figure highlights the critical differences between successful healing and the delayed healing pattern characteristic of current DES, illustrating the therapeutic paradox in which antiproliferative drugs that prevent restenosis simultaneously impair endothelial regeneration.

## 4. Extracellular Vesicles in Regulation of Endothelial Function and Regeneration

### 4.1. Endothelial Cell-Derived EVs: Master Regulators of Vascular Homeostasis

Endothelial cells are both the primary producers and primary targets of extracellular vesicles in the cardiovascular system. Even under physiological conditions, quiescent endothelium continuously secretes EVs that maintain vascular homeostasis through autocrine and paracrine signaling [71]. These endothelial cell-derived EVs (EC-EVs) contain a characteristic molecular signature rich in endothelial microRNAs, angiogenic proteins, and lipid mediators that promote vascular integrity, regulate permeability, maintain antithrombotic properties, and regulate inflammatory responses [72].

The miRNA cargo of EC-EVs demonstrates striking specificity. Endothelial “signature” miRNAs, including miR-126-3p/5p, miR-222-3p, miR-99b-5p, and members of the let-7 family, are consistently enriched in EC-EVs compared to other EV sources [72,73]. Among these, miR-126 stands out as the most abundant and functionally important endothelial miRNA, accounting for approximately 40% of the total miRNA content in some EC-EV preparations [74]. Encoded in an intron of the endothelial gene EGFL7, miR-126 serves as a key regulator of angiogenic signaling, vascular integrity, and endothelial inflammatory activation.

miR-126-enriched EC-EVs exhibit potent pro-angiogenic and barrier-stabilizing effects through coordinated targeting of multiple pathways. By suppressing SPRED1 and PIK3R2, two negative regulators of VEGF signaling, miR-126-3p potentiates VEGF-driven RAF/MEK/ERK and PI3K/AKT activation, which in turn promotes endothelial proliferation, migration, tube formation, and survival [75]. In parallel, miR-126-5p attenuates endothelial activation by targeting VCAM-1 and HMGB1, reducing leukocyte adhesion and inflammatory signaling [73,74].

EC-EVs also carry additional regulatory miRNAs, such as miR-143/145, which can be packaged into EVs from KLF2-activated endothelial cells and transferred to smooth muscle cells, stabilizing the contractile phenotype through inhibition of KLF4 and Elk-1 [76]. The let-7 family, also abundant in EC-EVs, exhibits dose- and context-dependent effects: at physiological levels, let-7b/c/f promotes endothelial barrier integrity and vascular organization by targeting negative regulators of angiogenesis such as STARD13 and FZD4, while overexpression can inhibit proliferation [71]. Together, these data position EC-EVs as central coordinators of vascular homeostasis, and Table 2 provides a structured overview of key miRNAs, source–target relationships, molecular targets, and functional effects.

### 4.2. Endothelial Progenitor Cell-Derived EVs: Specific Regenerative Mediators

Endothelial progenitor cells (EPCs) are bone marrow-derived precursors that can differentiate into mature endothelial cells and contribute to vascular repair. EPC-derived EVs (EPC-EVs) represent a particularly potent pro-regenerative EV subset with higher angiogenic potential compared to EVs derived from mature endothelial cells [74,83]. This superiority is attributed to a distinct miRNA and protein cargo optimized for vascular regeneration [68].

In preclinical models of ischemic stroke, miR-126-enriched EPC-EVs significantly reduce infarct size, increase angiogenesis and neurogenesis, attenuate apoptosis, and improve functional recovery [74]. Mechanistically, miR-126 in EPC-EVs downregulates SPRED1 and activates Raf/ERK signaling, promoting endothelial proliferation and migration at injury sites. Importantly, physiological stimuli can modulate EPC-EV activity: moderate exercise enhances the protective effects of circulating EPC-EVs through upregulation of the miR-126/BDNF/TrkB/Akt axis, suggesting that endogenous EPC-EV signaling could be exploited therapeutically or amplified [71,73].

In models of inflammatory vascular injury, EPC-EVs also exhibit potent anti-inflammatory and barrier-protective effects. EPC-EVs enriched with miR-126–3p/5p suppress LPS-induced inflammatory signaling by inhibiting HMGB1 and VEGFα-triggered permeability, while upregulating tight junction proteins and reducing endothelial leakage [71,83]. This combined ability to promote angiogenesis and simultaneously reduce inflammation makes EPC-EVs particularly attractive for stent-associated vascular regeneration.

Besides miR-126, EPC-EVs carry additional pro-angiogenic and cytoprotective factors, including miR-296 (PI3K-AKT/eNOS activation), miR-199a-3p (protection against ferroptosis and enhanced migration), VEGF, SDF-1, CXCR4, and eNOS itself [68,74]. This complex cargo allows EPC-EVs to activate multiple regenerative pathways in parallel, providing a plausible explanation for their superior efficacy compared to single-factor approaches.

### 4.3. Mechanisms of EV-Mediated Endothelial Regeneration

The regenerative effects of endothelium- and EPC-derived EVs result from several integrated mechanisms that restore a functional endothelial layer after vascular injury. These mechanisms include enhanced endothelial proliferation and migration, stabilization of barrier function, protection against apoptosis and oxidative stress, and modulation of inflammatory signaling [71,73,74].

Enhanced proliferation and migration. EV-derived miR-126 and other angiogenic miRNAs activate VEGF and PI3K/AKT signaling, accelerating endothelial cell cycle progression and directional migration. In vitro, EPC-EVs significantly enhance scratch wound closure and tube formation, widely used markers of angiogenic potential [71,73]. Notably, these proliferative effects occur without pathological endothelial activation, distinguishing EV-mediated regeneration from maladaptive proliferative responses.

Barrier protection and anti-permeability effects. EC and EPC-EVs increase endothelial barrier integrity by upregulating tight junction components and downregulating permeability-increasing pathways. miR-126-mediated inhibition of HMGB1 and VEGFα signaling reduces stress fiber formation and intercellular gap formation, while promoting the junctional localization of VE-cadherin and ZO-1 [74,83]. These effects offset the increased vascular leakage typically seen following stent-induced glycocalyx and endothelial injury.

Anti-apoptotic and cytoprotective signaling. By delivering miR-199a-3p, miR-296, VEGF, and eNOS, EPC-EVs reduce endothelial apoptosis and oxidative stress and promote survival in ischemic and inflammatory environments [68,74]. Activation of the PI3K-AKT and eNOS pathways further supports endothelial function by improving NO production and redox balance.

Immunomodulation. EPC-EVs reduce leukocyte adhesion and reduce endothelial inflammatory activation by modulating HMGB1 and other inflammatory mediators [71,83]. This immunomodulatory activity limits secondary endothelial damage and creates a more favorable environment for sustained regeneration.

Together, these mechanisms demonstrate that EV-based strategies not only accelerate endothelial coverage but also actively restore a functional, antithrombotic, and anti-inflammatory endothelium. This multilayered mechanism of action is directly relevant for the design of EV-inspired drug-coated devices.

### 4.4. Therapeutic Implications for Stent-Induced Vascular Injury

The biological properties of endothelium- and EPC-derived EVs directly address the fundamental challenges of stent-induced vascular injury. Endothelial loss, inflammatory activation, and exposure to antiproliferative drugs following stent deployment create a highly unfavorable environment for endothelial regeneration [51,54]. Current DES attempt to prevent restenosis through nonselective inhibition of cell proliferation, which inadvertently disrupts endothelial healing processes essential for long-term device safety [3].

EV-based approaches offer a fundamentally different paradigm: rather than suppressing all cellular activity, EVs can actively promote selective endothelial regeneration while regulating inflammation and smooth muscle cell responses. The feasibility of this approach has been demonstrated in recent preclinical studies. Hu et al. [17] showed that exosome-eluting stents accelerated re-endothelialization and reduced neointimal hyperplasia compared to conventional DES in rabbit models [17]. Zou et al. [18] developed enzyme-sensitive stents that released EPC-EVs in response to inflammatory signals (Lp-PLA2), resulting in a 42% reduction in neointimal thickness compared to bare-metal stents [18].

Critically, the dose–response relationship of EV therapy demonstrates a therapeutic window. Kesidou et al. [73] showed that EK-EVs most effectively promote angiogenesis at very low doses, while higher concentrations are less effective or even inhibitory [73]. This finding highlights the need for careful design of EV-mimetic stent coatings to achieve optimal local concentrations and the importance of controlled release kinetics and precise cargo loading. The specificity of EV-mediated effects represents another significant advantage. Unlike synthetic drugs that affect all proliferating cells equally, EV payloads (especially cell-type-enriched miRNAs such as miR-126) preferentially target endothelial cells due to the expression of their cognate mRNA targets [74]. This inherent selectivity could overcome a long-standing challenge in stent design by promoting endothelial healing without equivalently stimulating smooth muscle cell proliferation.

Table 3 systematically compares the endothelial regenerative effects of EVs derived from seven different cellular sources, including specialized populations such as endothelial cells, endothelial progenitor cells, mesenchymal stem cells, and cardiosphere-derived cells. The table reveals quantitative effects on proliferation (2–3-fold increase), migration, tube formation, and apoptosis reduction (50–60%), while also detailing the molecular mechanisms underlying these functional outcomes. This comprehensive comparison demonstrates that EV therapeutic efficacy is critically dependent on the cellular source and provides a rational framework for selecting optimal EV populations for device applications.

## 5. EVs and Smooth Muscle Cell Behavior: Phenotype Modulation and Implications for Device Design

The phenotypic plasticity of vascular SMCs presents both a challenge and an opportunity in the context of cardiovascular device development. Under physiological conditions, SMCs maintain a quiescent and contractile phenotype characterized by the expression of contractile proteins, including α-smooth muscle actin (α-SMA), smooth muscle myosin heavy chain (SM-MHC), smooth muscle protein 22α (SM22α), calponin, and smoothelin [64,65]. However, in response to vascular injury, such as stent implantation, SMCs can rapidly transition to a proliferative, migratory, synthetic phenotype characterized by decreased contractile protein expression and increased production of extracellular matrix, matrix metalloproteinases, proinflammatory cytokines, and EVs [65,67]. This phenotypic shift, initially adaptive for wound healing, leads to excessive neointimal hyperplasia and in-stent restenosis when dysregulated.

EVs play a crucial and context-dependent role in regulating SMC phenotype. The effects are critically dependent on the cellular origin of the EVs, their molecular cargo, and the physiological context of the recipient SMCs. Understanding these nuances is crucial for the rational design of EV-inspired cardiovascular devices that can selectively support endothelial regeneration while maintaining SMCs in a quiescent and contractile state.

### 5.1. Endothelial–SMC Communication Through EVs: The miR-143/145 Axis

One of the best-characterized examples of EV-mediated vascular homeostasis involves the transfer of miR-143/145 from endothelial cells to SMCs. The atheroprotective transcription factor Krüppel-like factor 2 (KLF2), which is upregulated in endothelial cells exposed to laminar shear stress, binds to the promoter region of the miR-143/145 cluster and induces its expression [76]. These microRNAs are then packaged into endothelial cell-derived EVs at extremely high concentrations; overexpression of KLF2 leads to a 30-fold enrichment of miR-143/145 in EVs compared to cellular supernatants. When these miR-143/145-enriched EVs are taken up by adjacent SMCs, they exert significant effects on maintaining the SMC phenotype. The miR-143/145 cluster targets transcription factors that promote the synthetic phenotype, specifically Krüppel-like factor 4 (KLF4) and Elk-1 [88,89]. By suppressing these dedifferentiation-promoting factors, miR-143/145 helps maintain SMCs in their contractile state, characterized by high expression of α-SMA, SM22α, and other contractile markers. Importantly, in co-culture experiments in which endothelial cells expressing KLF2 were paired with SMCs, recipient SMCs showed reduced proliferation, attenuated migration, and preserved contractile marker expression [76,90]. In vivo validation came from experiments showing that EVs derived from endothelial cells expressing KLF2 reduced atherosclerotic lesion formation in ApoE^−^/^−^ mice [76].

Recent studies have expanded the understanding of EK-SMC communication by identifying additional miRNAs involved in this crosstalk. Co-culture experiments with EV isolation following EK-SMC separation revealed that miR-539 was selectively enriched in EK-derived EVs during co-culture, while miR-582 was enriched in SMC-derived EVs. Functional analyses have shown that both microRNAs can modulate cellular phenotypes in both cell types, suggesting bidirectional communication pathways that fine-tune vascular homeostasis [81].

### 5.2. Context-Dependent Effects: Pathological and Homeostatic EVs

A critical concept emerging from recent research is that EVs derived from the same cell type can exert opposing effects on SMC behavior depending on the activation state of the parent cells. Under homeostatic conditions, endothelial cells continuously secrete “essential” EVs, which help maintain SMC contractility by reducing the expression of vimentin (a dedifferentiation marker) and inhibiting SMC proliferation and migration [91]. In contrast, when endothelial cells are exposed to atherogenic stimuli (such as 7-ketocholesterol, oxidized LDL, or impaired efflu), they secrete “pathological” EVs with significantly different cargo composition. These pathological EVs increase vimentin expression and promote SMC proliferation and migration, contributing to neointimal formation [73,91].

The molecular mechanisms underlying these context-dependent effects involve multiple pathways. Impaired flow, particularly at arterial bifurcations and atherosclerosis-prone regions, activates MAPK signaling in endothelial cells, leading to increased EV secretion. This impaired flow-induced EK-EVs promotes inflammatory polarization of macrophages, creating a pro-atherogenic microenvironment. Conversely, miR-34c-5p enriched in ECs under laminar flow could induce the conversion of M1 macrophages into M2 macrophages [92].

### 5.3. Mesenchymal Stem Cell-Derived EVs: Therapeutic Potential for Restenosis

Mesenchymal stem cell-derived EVs (MSC-EVs) have emerged as particularly promising candidates for the prevention of restenosis due to their ability to promote endothelial regeneration while simultaneously inhibiting SMC proliferation/migration. Numerous preclinical studies have demonstrated the efficacy of MSC-EVs in vascular injury models. In a pioneering study, adipose-derived MSC-EVs (ADMSC-EVs) significantly reduced intimal hyperplasia in a mouse vascular graft model (45 ± 9.0 μm compared to 26 ± 8.4 μm in controls, *p* < 0.05). The mechanisms are related to inhibition of VSMC proliferation and migration in vitro, decreased macrophage infiltration, and decreased expression of inflammatory cytokines (IL-6 and MCP-1). More importantly, ADMSC-EVs downregulated Akt, ERK1/2, and p38 phosphorylation in vascular graft models; these pathways are known to promote SMC proliferation [93].

Similar results were obtained in a rat carotid artery balloon injury model, where exosomes derived from bone marrow MSCs significantly inhibited neointimal hyperplasia through activation of ERK1/2 signaling [94]. Therapeutic efficacy was partially mediated through exosomal miR-125b, which targets myosin 1E (Myo1e), thereby suppressing SMC proliferation and migration [95]. Engineered approaches have further enhanced MSC-EV therapeutic potential; for example, EVs derived from bone marrow MSCs showing AT2R overexpression showed superior efficacy in preventing restenosis by promoting endothelial cell proliferation/migration while simultaneously inhibiting SMC proliferation/migration and preventing injury-induced phenotypic transformation [20].

### 5.4. EV-Mediated SMC Migration: A Double-Edged Sword

While excessive SMC migration contributes to neointimal hyperplasia, controlled SMC migration is essential for proper vascular repair. Recent mechanistic studies have revealed that sEVs can promote SMC migration through the presentation of extracellular matrix components, particularly collagen VI. In the presence of fibronectin in the ECM, VSMCs secrete polarized sEVs via filopodia via upregulation of the β1 integrin/FAK/Src and Arp2/3-dependent branched actin pathways. These sEVs induce the formation of focal adhesions at the cell periphery, switching the leading edge from protrusion activity to contractile mode to propel the cell body. Importantly, collagen VI loading in sEVs is indispensable for triggering focal adhesion formation and directed cell invasion [96]. This mechanism offers a potential therapeutic target to specifically modulate VSMC invasive activity during vascular repair without globally suppressing cellular function.

### 5.5. Implications of EV-Inspired Device Design

The evolving understanding of EV-mediated SMC regulation provides several fundamental principles for designing next-generation cardiovascular devices:

First, the cellular source of EVs is crucial. EVs derived from healthy, flow-stimulated endothelial cells or mesenchymal stem cells offer superior therapeutic profiles compared to EVs derived from activated inflammatory cells. Device surfaces can be designed to deliver such beneficial EVs or to release factors that stimulate local production of homeostatic EVs.

Second, miRNA loading represents a tunable parameter. The miR-143/145 cluster stands out as particularly promising for maintaining SMC contractility while supporting endothelial function. Devices can be designed to release synthetic or naturally derived EVs enriched with these specific microRNAs, creating a local microenvironment that promotes proper healing.

Third, the dose–response relationship is critical and likely non-linear. Just as EK-EVs show the highest efficacy at ultralow doses for endothelial regeneration [73], optimal SMC phenotype modulation may require careful titration of EV presentation or release kinetics.

Fourth, selective targeting can be achieved through the inherent targeting properties of EVs. Preferential uptake of certain EVs by endothelial cells compared to SMCs via surface integrins, tetraspanins, and other recognition molecules could enable cell-type-selective effects even when both cell types are present in the injured vessel wall.

Finally, the dynamic nature of EV-mediated communication suggests that devices should support the temporal evolution of healing rather than provide static suppression. Unlike antiproliferative drugs, which create a “frozen” healing state, EV-inspired devices can facilitate progression through the appropriate stages of healing: early inflammation resolution, selective endothelial regeneration, and ultimately restoration of the SMC contractile phenotype to prevent late excessive proliferation.

These principles position EV-inspired approaches not as simple alternatives to existing pharmacological strategies, but as fundamentally different paradigms that work alongside, rather than against, the body’s natural healing mechanisms. The following sections will examine how EVs regulate inflammation and how these biological insights are being translated into practical device technologies.

The differential effects of EVs from various cellular sources on SMC phenotype are detailed in Table 4, which compares the effects on contractile markers (α-SMA, SM22α, SM-MHC), proliferation, migration, and overall phenotypic status. The table particularly highlights context-dependent effects: Healthy endothelial cell-derived EVs maintain the contractile SMC phenotype via the miR-143/145 axis, whereas EVs originating from impaired flow or atherogenic conditions favor the synthetic phenotype. This phenotypic dichotomy, combined with quantitative data showing a 42% reduction in neointimal hyperplasia in MSC-EVs, provides a mechanistic rationale for selective EV-based therapeutic strategies that target pathological SMC proliferation while preserving physiological contractile function.

**Figure 3:**
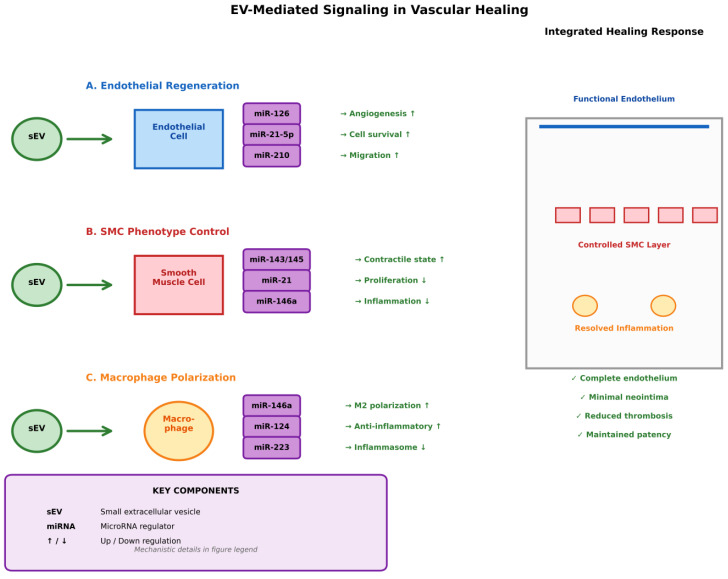
EV-mediated signaling pathways in vascular healing. Small EVs (sEVs) trigger coordinated repair responses by carrying therapeutic payload to recipient cells. (Panel **A**) Endothelial regeneration: miR-126 promotes angiogenesis (via SPRED1/PIK3R2 targeting), miR-21-5β increases cell survival (via PDCD4), and miR-210 facilitates migration (via Efna3). (Panel **B**) SMC phenotype control: miR-143/145 maintains contractile state (via KLF4/5), miR-21 limits proliferation (via PTEN), and miR-146α reduces inflammation (via NF-κB). (Panel **C**) Macrophage polarization: miR-146α promotes M2 polarization (via TRAF6/IRAK1), miR-124 enhances anti-inflammatory signaling (via C/EBPα), and miR-223 suppresses inflammasome (via NLRP3). The right panel shows the integrated healing response with functional outcomes. Arrows indicate upregulation (↑) or downregulation (↓). EV, extracellular vesicle; miR, microRNA; SMC, smooth muscle cell; sEV, small extracellular vesicle. Cell types (green sEVs, blue ECs, red SMCs, yellow macrophages), miRNA cargo (purple boxes).

Figure 3 provides an integrated visualization of EV-mediated intercellular communication networks in vascular healing and illustrates how endothelial cells, mesenchymal stem cells, and endothelial progenitor cells release EVs containing specific cargo (miRNAs, proteins, growth factors) that coordinate vascular repair by acting on target cells (regenerative endothelial cells, SMCs, and macrophages). The figure highlights the molecular mechanisms underlying these cell-type-specific effects, including the miR-126-SPRED1/PIK3R2 axis in endothelial regeneration, the miR-143/145-KLF4/Elk-1 pathway in maintaining the SMC phenotype, and miR-125b-mediated inhibition of SMC proliferation. This systems-level view reveals the paradigm shift from pharmacological suppression to biological regulation that underlies the EV-based device concept.

## 6. Immunomodulation and Inflammation Resolution: EVs as Central Coordinators

The inflammatory response following stent implantation is a critical determinant of long-term clinical outcomes. Acute inflammation is essential for the initiation of repair, while persistent or dysregulated inflammation leads to neointimal hyperplasia, late stent thrombosis, and neoatherosclerosis. EVs have emerged as central coordinators of the inflammatory microenvironment, possessing the capacity to either amplify or promote resolution of pathological inflammation depending on their cellular origin, molecular charge, and the context in which they act [13,98].

### 6.1. Macrophage Polarization: The M1/M2 Paradigm

Macrophages represent the most prevalent immune cell population infiltrating the damaged vessel wall following stent implantation. These highly plastic cells can adopt different polarization states along a spectrum, with M1 (“classically activated”) and M2 (“alternatively activated”) phenotypes representing functional endpoints. M1 macrophages stimulated by interferon-γ (IFN-γ) or lipopolysaccharide (LPS) promote inflammation, phagocytosis of pathogens, and cytotoxic responses by secreting proinflammatory cytokines, including tumor necrosis factor-α (TNF-α), interleukin-1β (IL-1β), IL-6, and IL-12. In contrast, M2 macrophages polarized by IL-4 or IL-10 produce anti-inflammatory mediators such as IL-10 and transforming growth factor-β (TGF-β), facilitate tissue repair, promote angiogenesis, and support ECM remodeling. The balance between M1 and M2 macrophages critically determines healing outcomes after vascular injury. Persistent M1 activation contributes to chronic inflammation, deficient endothelialization, and progressive neointimal thickening. Conversely, a timely transition to M2-dominant inflammation promotes resolution of inflammation, endothelial regeneration, and appropriate vascular remodeling. EVs play a key role in regulating this phenotypic transition [99].

### 6.2. MSC-Derived EVs: Master Regulators of M2 Polarization

Mesenchymal stem cell-derived EVs have demonstrated a potent immunomodulatory capacity, particularly in promoting M2 macrophage polarization. Numerous preclinical studies have shown that MSC-EVs reduce proinflammatory cytokine expression (IL-1β, IL-6, TNF-α) and increase anti-inflammatory mediators (IL-10, TGF-β) in both tissue and serum [39,100]. At the cellular level, MSC-EVs increase the expression of M2 markers, including Arginase-1 (Arg1) and CD206, while decreasing M1 markers, such as inducible nitric oxide synthase (iNOS), CD86, CD11b, and CD11c [100,101]. These effects have been confirmed in multiple experimental models. In myocardial infarction models, MSC-EVs promoted M2 polarization in both cardiac tissue and cultured macrophages exposed to hypoxia or LPS [102]. Intrapericardial delivery of cardiosphere-derived cell EVs in a porcine myocardial infarction model significantly increased the Arg1/iNOS ratio in pericardial fluid and circulating M2 monocytes (CD14^+^, CD163^+^) in peripheral blood 24 h after treatment [87]. These findings suggest that EVs may exert both local and systemic immunomodulatory effects.

Mechanistically, MSC-EV-mediated immunomodulation involves multiple pathways. The EV payload contains specific miRNAs that target inflammatory signaling cascades, membrane-bound proteins that interact with macrophage surface receptors, and bioactive lipids that regulate inflammatory mediator production. The therapeutic potential of this approach has been demonstrated in vascular injury models, where ADMSC-EVs significantly reduced macrophage infiltration and inflammatory cytokine expression (IL-6 and MCP-1) while reducing neointimal hyperplasia [93].

### 6.3. Context-Dependent Effects: EV Origin Matters

Importantly, not all EVs promote inflammation resolution; some actively propagate inflammatory cascades. The functional impact of EVs is critically dependent on the activation state of their host cells. M1 macrophage-derived EVs often carry pro-inflammatory cargo that can sustain inflammatory signaling in recipient cells. However, emerging evidence reveals an unexpected complexity: even M1-derived EVs can paradoxically promote M2 polarization in recipient macrophages under certain conditions, suggesting context-dependent plasticity in EV function [40].

M2 macrophage-derived EVs generally exhibit anti-inflammatory effects. These EVs are enriched with molecules that suppress pro-inflammatory enzymes and cytokines (IL-12, TNF-α) while delivering anti-inflammatory factors (IL-10, TGF-β) [103]. In cardiovascular contexts, M2-EV cargo may include mitochondria, which are transferred to injured cardiomyocytes via EV-dependent pathways, thereby inhibiting oxidative stress and promoting cellular recovery. M2-EVs also contain LncRNA AK083884, which regulates the PKM2/HIF-α and SOCS2/STAT6 pathways to inhibit glycolysis and promote further M2 polarization, creating a positive feedback loop that amplifies anti-inflammatory responses [104].

### 6.4. Engineered EVs for Targeted Immunomodulation

Recent advances have focused on engineering EVs to enhance their immunomodulatory properties and targeting capabilities. Surface functionalization strategies allow EVs to localize specifically to sites of vascular injury or inflammation. For example, M2 macrophage-derived EVs fused with platelet membranes demonstrated improved targeting to atherosclerotic plaques due to the inherent affinity of platelet membrane components for activated endothelium and exposed extracellular matrix [11,105]. This biomimetic approach combines the anti-inflammatory payload of M2-EVs with the site-specific targeting properties of platelet-derived vesicles.

Similarly, EVs derived from endothelial cells subjected to laminar shear stress (LSS-EVs) demonstrated improved therapeutic efficacy when modified with hyaluronic acid (HA) targeting ligands. HA specifically binds to CD44, which is highly expressed by macrophages in atherosclerotic plaques. LSS-EVs enriched with miR-34c-5p promoted macrophage repolarization from M1 to M2 by targeting TGIF2 and activating the TGF-β-Smad3 signaling pathway. HA-modified LSS-EVs (HA@LSS-EVs) showed remarkable specificity for atherosclerotic lesions and potent therapeutic effects both in vitro and in vivo [8].

### 6.5. EVs and Thrombosis: A Double-Edged Sword

In addition to their role in inflammation, EVs also critically impact thrombotic complications, a major cause of late stent failure. EVs can carry procoagulant factors, particularly tissue factor (TF), which initiates the extrinsic coagulation cascade. Endothelial cell-derived EVs can interact with platelets and monocytes, affecting endothelial dysfunction, atherosclerotic plaque destabilization, and thrombus formation [13]. In pathological conditions, high circulating EV-TF activity has been associated with increased thrombotic risk and disease severity [106].

However, EVs also possess antithrombotic properties depending on the cargo they carry. Platelet-derived EVs (PEVs) carrying specific miRNAs (miR-223, miR-320b) can potentially reduce thrombotic risk by regulating endothelial inflammation and oxidative stress through inhibition of MAPK and NF-κB signaling pathways [79]. The balance between pro-thrombotic and anti-thrombotic EV populations in the circulation may influence the risk of late stent thrombosis, an area requiring further investigation.

### 6.6. Temporal Dynamics of EV-Mediated Inflammation

In the acute phase following stent implantation, platelet-derived EVs increase within the first 24–48 h and are associated with local inflammatory activity and subsequent risk of restenosis [107]. As healing progresses, experimental models suggest that EVs released by endothelial progenitor cells and alternatively activated (M2) macrophages exhibit greater pro-repair function, shifting the inflammatory environment toward resolution by promoting endothelial cell survival, angiogenesis, and vascular remodeling [108]. In the chronic phase (weeks to months), the persistent release of pro-inflammatory EVs, which may be triggered by polymer-induced foreign body reactions or incompletely healed tissue, can perpetuate maladaptive inflammation. DES explants showed chronic infiltration of eosinophils and lymphocytes, suggesting ongoing hypersensitivity reactions mediated in part by EV-dependent intercellular communication [54]. Understanding these temporal dynamics could inform strategies for delivering EV-based therapies or EV-inspired signals at optimal time points to direct healing.

### 6.7. Implications for Device Design

The central role of EVs in immunomodulation offers many opportunities for next-generation device design:

First, devices can be designed to release anti-inflammatory EVs or EV-mimetic nanoparticles loaded with M2-polarizing cargo (specific miRNAs, anti-inflammatory proteins). Release kinetics can be designed to match the natural inflammation timeline, with higher release during the acute inflammation phase, when modulation is most critical.

Second, device surfaces can be designed to modulate local EV production; This promotes the release of homeostatic EVs from adherent endothelial cells while minimizing the production of pro-inflammatory EVs from activated immune cells. Surface topography, chemistry, and mechanical properties all influence cellular EV secretion patterns.

Thirdly, EV capture strategies can selectively isolate pro-inflammatory EVs while allowing beneficial EVs to circulate and communicate. Affinity ligands targeting specific EV surface markers (e.g., inflammatory cell-derived tetraspanins) can be incorporated into device coatings.

Fourthly, by incorporating biomimetic EV components (especially M2-polarizing miRNAs or anti-inflammatory proteins) into their structures, devices can create surfaces that consistently deliver immunomodulatory signals without requiring intact EV structures. These EV-centric approaches represent a paradigm shift from current immunosuppressive strategies. Rather than suppressing immune function generally (as with corticosteroids) or nonselectively inhibiting cell proliferation (as with sirolimus/paclitaxel), EV-inspired devices will actively direct the immune response toward productive healing and promote timely inflammation resolution while preserving tissue repair and regeneration capacity.

The immunomodulatory effects of EVs derived from various cellular sources are systematically compared in Table 5, with particular emphasis on their ability to direct M1 → M2 macrophage polarization. The table documents quantitative changes in polarization markers (increased Arg1 and CD206; decreased iNOS and CD86) and inflammatory cytokines (decreased TNF-α, IL-1β, and IL-6; increased IL-10 and TGF-β). Of particular interest are engineered approaches such as hyaluronic acid-modified laminar shear stress EVs (HA@LSS-EVs), which enable targeted delivery to CD44+ macrophages in atherosclerotic plaques, and M2-EV/platelet membrane hybrids, which enhance plaque targeting. These strategies demonstrate how EV surface engineering can enable spatial control over immunomodulation, a critical capability for next-generation cardiovascular devices.

## 7. From Biology to Technology: EV-Inspired Drug-Coated Devices

Previous sections have outlined the biological rationale for EV-based approaches to vascular healing: EVs selectively promote endothelial regeneration, modulate SMC phenotype toward contractile states, regulate inflammation resolution, and drive macrophage polarization, all processes that current DES suppress rather than control. Translating these biological insights into practical medical devices represents a challenging yet achievable engineering challenge. Recent pioneering studies have demonstrated proof of concept for EV-eluting stents and highlighted fundamental design principles for next-generation devices [17,18].

### 7.1. Exosome-Releasing Stents: Proof of Concept

A groundbreaking 2021 study by Hu and colleagues demonstrated the feasibility and therapeutic potential of exosome-eluting stents (EES) [17]. The team developed stents coated with mesenchymal stem cell-derived exosomes embedded in a polymer matrix sensitive to reactive oxygen species (ROS). The design addressed two critical challenges: first, enabling the controlled release of exosomes in response to the inflammatory microenvironment; second, preserving exosome bioactivity during the coating process and storage.

The ROS-sensitive release mechanism proved particularly ingenious. Vascular damage and inflammation generate high levels of reactive oxygen species at the implantation site [110]. The polymer matrix degrades, particularly in this ROS-rich environment, releasing intact exosomes with preserved biological activity. This “smart release” trigger prevents premature depletion of the therapeutic payload while ensuring maximum exosome delivery when and where it is needed most. In rat models of renal ischemia–reperfusion injury followed by abdominal aortic stenting, EES demonstrated superior performance compared to both bare-metal stents (BMS) and conventional DES. By 28 days post-implantation, EES demonstrated significantly accelerated re-endothelialization and nearly complete endothelial coverage compared to DES. Neointimal hyperplasia was reduced compared to BMS, and EES provided a reduction in neointimal thickness comparable to or better than DES, but without the delayed healing and incomplete endothelialization that characterize DES [17].

EES demonstrated systemic regenerative effects beyond the stent. Released exosomes promoted muscle regeneration by traveling to ischemic hindlimb tissue [17]. This dual functionality (local vascular healing and distant tissue repair) represents a paradigm shift from current stents, which focus solely on preventing restenosis at the device site.

### 7.2. Enzyme-Sensitive EV Release: Targeted Delivery

Building on the EES concept, Zou et al. developed a more advanced bioresponsive system involving enzyme-triggered exosome release. Their design utilized lipoprotein-associated phospholipase A2 (Lp-PLA2), an enzyme elevated in atherosclerotic plaques and inflammatory vascular lesions. Lp-PLA2 levels are associated with cardiovascular risk and are particularly elevated in areas of plaque instability. The engineering strategy involved encapsulating MSC-derived exosomes within liposome-based multivesicular compartments that were transported into vascular stents. The liposomal vesicles were designed with phospholipid compositions sensitive to Lp-PLA2 hydrolysis. Upon contact with Lp-PLA2 in the local vascular microenvironment, the enzyme cleaves phospholipid bonds, destabilizing the vesicles and triggering exosome release [18]. This approach achieves several key objectives:

First, spatial specificity: exosomes are preferentially released in regions with high Lp-PLA2 levels, corresponding to areas of greatest pathological need. Second, temporal control: release kinetics are aligned with disease activity, resulting in faster release in settings with high inflammation. Third, dose proportionality: the magnitude of exosome release is proportional to the Lp-PLA2 concentration, creating a self-regulating therapeutic system.

Exosomes are designed to address a critical pathological process: defective efferocytosis, the clearance of apoptotic cells by macrophages. Failure of efferocytosis in atherosclerotic plaques beneath stents perpetuates chronic inflammation and drives plaque progression. MSC-derived exosomes regulate efferocytosis through multiple signaling pathways, including SLC2a1, STAT3/RAC1, and CD300a, while regulating foam cell formation through CD36-mediated pathways. In rat models of mal-efferocytosis, enzyme-sensitive, exosome-releasing stents significantly increased apoptotic cell clearance rates and reduced neointimal thickness by approximately 42% compared to bare-metal stents [18]. The multi-mechanistic effect of exosomes, simultaneously improving efferocytosis, reducing foam cell formation, and regulating inflammation, represents the advantage of biologically complex therapies over single-target drugs.

### 7.3. EV-Inspired Device Engineering Framework: From Biology to Design Rules

The biological principles outlined above can be translated into a set of design rules for next-generation cardiovascular devices. Instead of acting as inert scaffolds or simple drug depots, EV-inspired devices should mimic four key features of endogenous vesicle signaling: sensing, signaling, scaffolding, and synergy [12,15].

Sensing; microenvironment-responsive release: Natural EVs are produced and released in response to defined cues such as hypoxia, oxidative stress, and inflammatory activation, and are primarily trafficked to sites of endothelial activation or matrix exposure [22,41]. Exosome-releasing stents are beginning to approximate this behavior by using ROS-sensitive or enzyme-sensitive polymers that couple the local inflammatory state to vesicle release [17,20]. Therefore, a key design goal for EV-inspired coatings is to incorporate chemical or enzymatic sensors (e.g., ROS-cleavable linkers, Lp-PLA2-sensitive liposomes) that dynamically adjust their release kinetics to the evolving biology of the damaged vessel wall [17,20].

Signaling; encoded miRNA and protein “instruction sets”: EVs provide compact “instruction sets” in the form of microRNAs, proteins, and lipids that reprogram endothelial cells, smooth muscle cells, and macrophages toward healing-supportive phenotypes [7,14,15]. In contrast, current drug-eluting stents rely on broad antiproliferative agents that indiscriminately suppress the cell cycle [3]. EV-inspired devices should prioritize phenotype-regulating signals, such as miR-126-enriched cargo to support endothelial regeneration, miR-143/145 clusters to stabilize the contractile SMC phenotype, and miR-146a or similar immune-regulatory miRNAs to promote M2 macrophage polarization [39,75,76,95]. This reframes device pharmacology from simple inhibition toward vascular niche reprogramming.

Scaffold; ECM-mimetic physical and biochemical cues: EVs move within and on the extracellular matrix, binding to collagen, proteoglycans, and glycosaminoglycans, affecting their local concentration, diffusion, and uptake [12,29,47]. ECM-mimetic coatings based on recombinant collagen III or nanofibrous scaffolds demonstrate how biomimetic architectures can regulate cell adhesion, shear sensing, and EV capture at the device–tissue interface [18,38]. Integrating EV-like signaling into such ECM-mimetic scaffolds enables the development of devices that provide both guiding biochemical cues and favorable mechanical microenvironments, supporting coordinated endothelial and SMC repair rather than simply suppressing cell proliferation [17,19].

Synergy; combining local vascular healing with systemic regeneration: A distinctive feature of EV-based approaches is their ability to combine local and systemic effects. In preclinical models, exosome-eluting stents not only accelerate re-endothelialization and reduce neointimal hyperplasia but also deliver regenerative signals to distant ischemic tissues [17]. This local-systemic synergy is not present in current drug-eluting platforms [1,3]. Designing devices that exploit this property (by controlling vesicle dose, release direction, and biodistribution) could transform stents from purely local interventions into systemic modulators of cardiovascular repair [12,19].

### 7.4. Biomimetic Coatings: Lessons from ECM and EV Biology

Beyond direct exosome delivery, researchers have developed biomimetic coatings that incorporate principles derived from ECM and EV biology. Chen and colleagues created a “dual bionic” ECM-mimetic coating that combines structural mimicry (nanofiber architecture) with component mimicry (recombinant humanized collagen type III). The nanofiber network mimics the three-dimensional fibrous structure of native ECM, creating a biomimetic topography that influences cellular behavior while providing a scaffold for enhanced protein immobilization [57].

The rhCollagen III component provides multiple functions: anti-coagulation, anti-inflammatory properties, promotion of EC proliferation, and induction of a contractile SMC phenotype. In vivo assays have demonstrated superior antithrombosis capacity, accelerated re-endothelialization, and inhibition of SMC proliferation compared to controls [18]. The coating essentially recreates key functional aspects of the healthy vascular ECM, an environment in which endothelial cells thrive and SMCs maintain quiescence.

### 7.5. Double-Exome Coatings: Complementary Functions

Recognizing that different EV populations offer complementary benefits, researchers have developed double exosome coating strategies. By proportionally grafting exosomes from different cellular sources onto device surfaces using biotin–avidin interactions, these coatings simultaneously address multiple therapeutic targets. Double exosomes facilitated the formation of functionally intact endothelial layers, inhibited macrophage adhesion, suppressed inflammation, promoted SMC phenotypic conversion to contractile states, reduced thrombosis, and reduced neointimal thickness [20,111]. Molecular mechanisms include upregulation of eNOS expression and downregulation of NOX1/NOX4 to reduce oxidative stress and restore endothelial function. Exosomes also upregulate SLC29a1 and SLC2a1, while downregulating CD300a, CD36, and Lp-PLA2, promoting efferocytosis and inhibiting inflammation [111]. This multi-targeted approach addresses the complexity of vascular pathology more comprehensively than single-mechanism interventions.

### 7.6. Nanoparticle-Based Delivery Platforms

The challenges of large-scale exosome production and standardization have spurred the development of biomimetic nanoparticles that replicate essential EV functions without requiring intact vesicles. Synthetic nanoparticles can be engineered to carry specific EV-derived cargoes (miRNAs, proteins, lipids) while offering advantages in terms of production scalability, batch-to-batch consistency, loading efficiency, and storage stability [25].

Many nanoparticle platforms hold promise for cardiovascular applications. Lipid-based nanoparticles, particularly when their surfaces are modified with degradable polymers, enable targeted delivery of biomolecular therapeutics with high specificity and minimal off-target effects [111,112]. Compared to traditional DCBand DES, which lack cell-type specificity, nanoparticle-based delivery can be designed to be preferentially taken up by endothelial cells rather than SMCs, thus achieving the selective therapeutic effects observed in native EVs [112,113].

Enzyme-responsive nanoparticle systems offer particularly advanced control over drug release. By incorporating enzyme-cleavable moieties into the main chains or side groups of nanoparticle structures, researchers can create materials that are specifically degraded in response to disease-related enzymes such as matrix metalloproteinases, sPLA2, or cathepsins [111]. This degradation releases the therapeutic payload to sites of enzyme activity, which generally correspond to areas of greatest pathological need. This spatial and temporal control enhances therapeutic efficacy while minimizing systemic exposure and off-target effects.

### 7.7. Design Principles for Next-Generation Devices

The collective experience gained from EV-eluting stents, biomimetic coatings, and nanoparticle systems reveals several fundamental design principles for next-generation cardiovascular devices:

1. Controlled Release is Essential: Simple surface coating with EVs or nanoparticles carries the risk of rapid burst release and premature depletion. Responsive release mechanisms triggered by ROS, inflammatory enzymes, or other disease-related signals enable sustained therapeutic delivery tailored to healing kinetics.
2. Bioactivity Preservation: EVs are complex biological entities that can lose their functionality during processing, storage, or exposure to in vivo physiological conditions. Protective matrices, lyophilization strategies, and appropriate storage conditions are critical for maintaining therapeutic efficacy.
3. Multi-Mechanism of Action is Advantageous: Vascular healing involves multiple interconnected processes (endothelial regeneration, SMC quiescence, resolution of inflammation, prevention of thrombosis). Therapies that simultaneously target multiple targets, such as natural EVs, are more likely to be successful than single-mechanism drugs.
4. Cell Type Selectivity: Ideal devices support endothelial proliferation while maintaining SMC contractility. Natural EVs achieve this through cell type-specific cargo and receptor-mediated targeting. Synthetic systems should incorporate similar selectivity mechanisms.
5. Manufacturing Scalability: Clinical translation requires reproducible and scalable manufacturing. This supports either standardized exosome production protocols with stringent quality control or a shift to fully synthetic biomimetic nanoparticles that replicate core EV functions.
6. Regulatory Pathway Clarity: EV-based devices create regulatory complexity by crossing the boundary between biologics and devices. Early engagement with regulatory agencies and clear definition of critical quality attributes are essential for clinical development.

### 7.8. Challenges and Opportunities

#### 7.8.1. Scientific and Technical Challenges

The clinical opportunity for EV-inspired cardiovascular devices is significant. Current drug-eluting stents (DES), despite their effectiveness in reducing one-year restenosis, do not demonstrate a long-term outcome advantage over bare-metal stents after the first year, largely due to delayed healing and late adverse events [3]. EV-inspired devices that actively promote vascular repair rather than broadly suppressing cellular activity could fundamentally alter this risk–benefit profile, reducing the need for long-term dual antiplatelet therapy and lowering the risk of late-stage stent thrombosis.

Furthermore, the regenerative capacity demonstrated by EV-eluting stent platforms, where released EVs support tissue repair beyond the stent site, suggests applications extending beyond simple restenosis prevention. Patients with critical limb ischemia, multi-vessel coronary artery disease, or complex vascular lesions may particularly benefit from devices that combine mechanical support with biologically guided regenerative signaling.

The following discussion examines the remaining challenges and future perspectives for EV-inspired cardiovascular devices. Three proof-of-concept studies demonstrating the feasibility of EV-releasing stents are systematically compared in Table 6, summarizing their experimental models, EV sources, smart release mechanisms, and quantitative results. As shown, Hu et al. [17] achieved accelerated re-endothelialization using polymer coatings sensitive to reactive oxygen species (ROS), while Zou et al. [20] reported a 42% reduction in neointimal thickness via EV release triggered by lipoprotein-associated phospholipase A2 (Lp-PLA2). Collectively, these studies provide proof-of-principle that EV-based devices can regulate smooth muscle cell and inflammatory responses while promoting endothelial regeneration; effects that are difficult to achieve with conventional single-drug approaches.

#### 7.8.2. Manufacturing and Regulatory Challenges

Despite encouraging proof-of-concept results, significant manufacturing and regulatory challenges need to be addressed before EV-inspired devices can be translated into routine clinical practice. Clinical-scale EV production requires significant cell culture capacity, robust and standardized purification workflows, and reproducible quality control strategies. Batch-to-batch variability in EV composition can affect therapeutic consistency, while long-term storage stability remains a limitation, as most current approaches rely on frozen storage, complicating clinical logistics and distribution.

However, these challenges are not insurmountable. The pharmaceutical and biotechnology sectors have successfully scaled up other cell-derived therapies and provided a framework that can be adapted for EV production. In parallel, EV-mimicking nanoparticle systems designed to deliver defined cargo compositions can help overcome some of the limitations associated with naturally occurring EVs while preserving essential biological functions. From a regulatory perspective, clear classification, identification of critical quality characteristics, and establishment of standardized release criteria will be essential to ensure safe, scalable, and compliant clinical translation.

**Figure 4:**
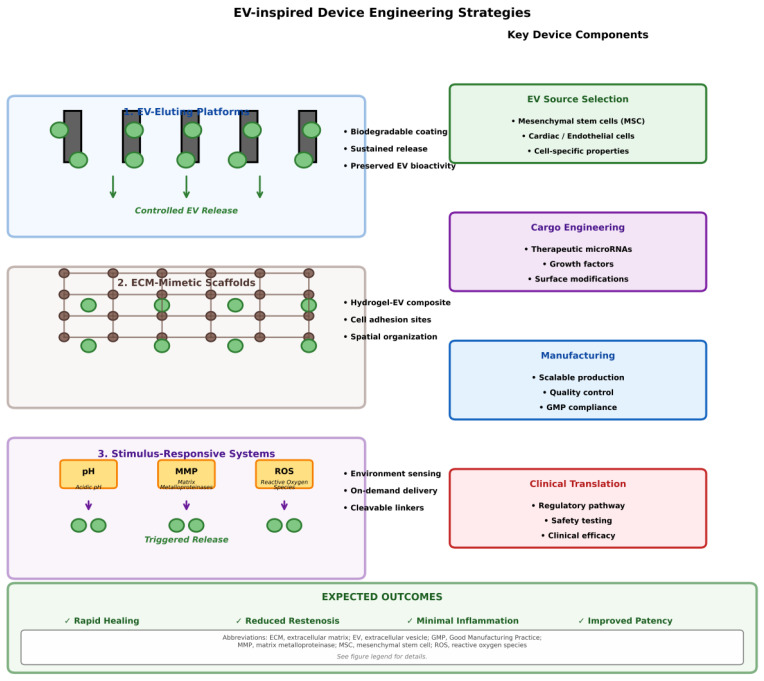
EV-inspired design principles for cardiovascular device coatings. The left panel illustrates three engineering strategies: (1) EV-releasing platforms with biodegradable coatings that enable controlled release while preserving bioactivity; (2) ECM-mimicking scaffolds providing cell adhesion sites and spatial organization to hydrogel-EV composites; (3) Stimulus-responsive systems triggered by injury-related cues (pH, matrix metalloproteinases, reactive oxygen species). The right panel summarizes the key components: EV source selection (mesenchymal stem cells, cardiac/endothelial cells); cargo engineering (therapeutic microRNAs, growth factors, surface modifications); manufacturing (scalable production, quality control, GMP compliance); and clinical translation (regulatory pathway, safety testing, clinical efficacy). The bottom panel shows the expected outcomes: rapid healing, reduced restenosis, minimal inflammation, and improved patency. ECM, extracellular matrix; EV, extracellular vesicle; GMP, Good Manufacturing Practices. Color coding and symbols: EVs (green circles), stent struts (gray), ECM nodes (brown circles), stimulus sensors (yellow boxes), release direction (green and purple arrows), positive outcomes (green checkmarks, ✓). Engineering strategy panels distinguished by background colors (light blue, beige, light purple). Component boxes color-coded: EV source (green), cargo engineering (purple), manufacturing (blue), clinical translation (red).

Figure 4 provides a comprehensive schematic of the EV-eluting stent design and function. Panel A shows the cross-sectional structure showing the metal support core, ROS-sensitive polymer matrix, and embedded MSC-derived exosomes (10^11^–10^12^ per stent). Panel B details the smart release mechanisms, both ROS-triggered polymer degradation in inflammatory microenvironments and Lp-PLA2 enzyme-sensitive liposome destabilization; these mechanisms provide spatial and temporal control over exosome delivery. Panel C compares the in vivo performance between bare-metal stents (significant neointimal hyperplasia, incomplete healing), DES (reduced neointima but delayed re-endothelialization), and exosome-eluting stents (accelerated endothelial coverage, functional recovery, systemic regenerative effects). Quantitative data from proof-of-concept studies demonstrate that EV-based devices can achieve therapeutic goals that traditional approaches have failed to achieve: complete vascular healing without compromising long-term safety.

## 8. Future Perspectives and Translational Pathways

While the previous section summarized the current challenges and opportunities, this section focuses on the future scientific advances and translation pathways needed to bring EV-inspired cardiovascular devices into routine clinical practice.

### 8.1. Scientific Advances Needed

Further advances in EV-inspired cardiovascular device design will depend on a deeper mechanistic understanding of EV–cell interactions in the damaged vascular microenvironment. Key scientific priorities include elucidating how EV cargo composition, surface molecules, and release kinetics affect endothelial regeneration, smooth muscle cell phenotype, and immune modulation in a spatially and temporally controlled manner. A better understanding of EV biodistribution, uptake efficiency, and intracellular transport will be essential to enhance therapeutic sensitivity and efficacy.

Advances in EV characterization and standardization are also necessary. Robust analytical frameworks that correlate the physicochemical properties and cargo profiles of EVs with biological activity will facilitate the rational design and comparison of EV-based or EV-inspired platforms. In parallel, the development of predictive in vitro and in vivo models capturing clinically relevant vascular injury and healing dynamics will be critical for validating therapeutic mechanisms and optimizing device performance. Together, these scientific advances will enable more precise, reproducible, and mechanism-oriented EV-inspired interventions.

### 8.2. Translation and Implementation Pathways

In terms of translation, successful clinical implementation of EV-inspired devices will require the integration of scalable manufacturing strategies with regulatory-compliant quality systems. Clear definition of critical quality characteristics, batch release criteria, and stability requirements is essential to ensure reproducibility and safety. Compliance with Good Manufacturing Practices (GMP) standards and early engagement with regulatory authorities can help clarify product classification and streamline approval pathways.

Transition to clinical practice will also depend on appropriately designed studies and endpoints that go beyond traditional metrics such as restenosis rates. Metrics that capture endothelial healing, inflammation resolution, and long-term vascular function may better reflect the therapeutic goals of EV-inspired devices. As experience with bioactive coatings and combination products increases, lessons learned from previous generations of drug-eluting stents and cell-based therapies can shed light on study design, risk stratification, and post-market surveillance. Ultimately, realizing the full potential of EV-inspired cardiovascular technologies will require coordinated progress across scientific, manufacturing, regulatory, and clinical domains.

Key scientific and translational priorities that will shape the future development and clinical applications of EV-inspired cardiovascular devices are summarized in Table 7.

## 9. Conclusions

EV biology offers a mechanistic template for a next generation of drug-eluting cardiovascular devices that goes beyond selective antiproliferative drug delivery, focusing on the coordinated regulation of endothelial repair, smooth muscle cell phenotype, and inflammation resolution. The proof-of-concept of EV-inspired coatings demonstrates the possibility of cell-directed drug delivery that promotes healing; however, implementation will require pathway-knowledge-based design, scalable manufacturing with defined critical quality characteristics, regulatory approval, and clinical trials that capture both classical outcomes and the quality of vascular healing. If these hurdles are overcome, EV-inspired devices could help solve the long-standing problem of balancing restenosis prevention with long-term vascular restoration.

## Figures and Tables

**Table 1:** Classification and Characteristics of Extracellular Vesicles (MISEV23).

EV Type	Size Range	Biogenesis Pathway	Key Markers	Cargo	Primary Functions
Small EVs (Exosomes)	30–150 nm (<200 nm)	Endosomal origin: ESCRT-dependent ESCRT-independent (ceramide, lipid rafts) MVB fusion with the plasma membrane Extra-hard returns	Tetraspanins (CD9, CD63, CD81) ESCRT proteins (TSG101, Alix) HSPs (HSP70, HSP90) Flotillin	miRNAs (miR-126, miR-143/145, miR-125b) Proteins (growth factors, cytokines) Lipids (cholesterol, ceramide) mRNAs, lncRNAs	Intercellular communication Phenotype reprogramming Immunomodulation Regulation of angiogenesis Tissue regeneration
Large EVs	100–1000 nm (>200 nm)	Direct budding from the plasma membrane: Cytoskeletal reorganization Lipid redistribution Ca^2+^-dependent	Integrins (α5β1, αvβ3) Selectins CD40 ligand Tissue factor Membrane proteins	Surface receptors Enzymes (MMPs) Procoagulant factors Nucleic acids Cytoplasmic proteins	Coagulation Cell activation Matrix remodeling Signal transduction Regulation of thrombosis
Apoptotic Bodies	1–5 μm	Released during apoptosis: Membrane blebbing Cell lysis Caspase activation	Phosphatidylserine Annexin V Histones Fragmented DNA Thrombospondin	Nuclear particles Organelles Chromatin Cellular debris Oxidized lipids	Apoptotic cell clearance “Eat me” signals Immune response Efferocytosis Anti-inflammatory

**Table 2:** Important miRNAs in EV-Mediated Vascular Healing.

miRNA	Source Cell	Target Cell	Molecular Targets	Biological Effects	Key References
miR-126-3p/5p	Endothelial cells, EPCs	Endothelial cells	SPRED1, PIK3R2, VCAM-1, DLKC1	↑ Proliferation, ↑ Migration, ↑ Tube formation, ↑ VEGF signaling, ↓ Apoptosis, ↑ Barrier function	[77,78]
miR-143/145	Endothelial cells (KLF2-induced)	SMCs	KLF4, Elk-1, PDGF-Rα	↑ Contractile phenotype (α-SMA, SM22α, SM-MHC), ↓ Proliferation, ↓ Dedifferentiation	[79]
miR-125b	MSCs	SMCs	Myo1e	↓ Proliferation, ↓ Migration, ↓ Neointimal hyperplasia	[79]
miR-146a	MSCs	Macrophages	TRAF6, IRAK1, NF-κB pathway	↓ Inflammatory response, M1 → M2 polarization, ↓ TNF-α, ↓ IL-6	[80]
miR-539	Endothelial cells (co-culture)	SMCs	Multiple targets in proliferative pathways	Bidirectional EC-SMC communication, phenotype modulation	[81]
miR-223	Platelets, Leukocytes	Endothelial cells, Macrophages	MAPK, NF-κB, NLRP3	↓ Inflammation, ↓ Thrombosis, Anticoagulant effects	[79]
miR-320b	Endothelial cells	SMCs	MAPK signaling	↓ Thrombosis, Anti-inflammatory effects	[79]
miR-34c-5p	Laminar shear stress-EVs	Macrophages	TGIF2, TGF-β-Smad3 pathway	M1 → M2 repolarization, ↓ Atherosclerotic plaque inflammation	[79]
miR-296	EPCs	Endothelial cells	HGS (hepatocyte growth factor-regulated tyrosine kinase substrate)	↑ Angiogenesis, ↑ VEGFR2 signaling	[82]
miR-199a-3p	EPCs	Endothelial cells	Sema3A (semaphorin 3A)	↑ Angiogenesis, ↑ EC migration, ↓ Apoptosis	[82]

Symbols: ↑, increased; ↓, decreased; →, transition.

**Table 3:** EV Effects on Endothelial Cell Function.

EV Source	Key Load/Mechanisms	Functional Results	Molecular Effects	Main Studies
Endothelial Cell-EVs	miR-126 (highly enriched) eNOS VEGF Angiogenic proteins	↑ Proliferation (2–3-fold) ↑ Migration ↑ Tube formation ↑ Wound healing ↑ Barrier integrity	SPRED1/PIK3R2 inhibition VEGF-Akt-eNOS activation NO production ↑ Oxidative stress ↓	[77]
Endothelial Progenitor Cell-EVs	miR-126 (5–10-fold enriched) miR-296, miR-199a-3p VEGF, SDF-1, CXCR4 Pro-angiogenic factors	↑↑ Angiogenesis (enhanced) ↑ EC survival ↑ Vascular repair ↓ Apoptosis (50–60%) ↑ Re-endothelialization	Enhanced VEGFR2 signaling PI3K-Akt pathway activation Anti-apoptotic proteins ↑ Sema3A inhibition	[75]
MSC-EVs	Multiple miRNAs Growth factors Anti-inflammatory proteins Immunomodulatory factors	↑ Proliferation ↑ Migration ↓ Apoptosis ↑ Barrier function ↓ Oxidative stress	Multiple pathway activation ROS reduction Inflammatory cytokines ↓ eNOS expression ↑	[84]
Platelet-EVs	miR-223 Growth factors (VEGF, PDGF) Adhesion molecules P-selectin	Context-dependent: ↑ EC activation (acute) ↑ Angiogenesis (chronic) ↑ Barrier permeability	Early inflammatory signals Later angiogenic effects Dual pro/anti-inflammatory	[85]
AT2R-Overexpressing MSC-EVs	AT2R pathway activation Enhanced pro-angiogenic load Anti-inflammatory factors	↑↑ EC proliferation ↑↑ Migration ↑ Tube formation ↓ SMC proliferation (dual benefit)	AT2R-mediated signaling NO production ↑ VEGF signaling ↑ Selective EC production	[86]
Laminar Shear Stress-EVs (LSS-EVs)	Shear-sensitive microRNAs Atheroprotective factors KLF2-mediated load	↑ EC alignment ↑ Atheroprotection ↓ Inflammatory activation ↑ eNOS expression	Mechanosensitive pathways KLF2 activation Anti-inflammatory effects eNOS phosphorylation	[79]
Cardiosphere-Derived Cell-EVs	Cardioprotective proteins Pro-angiogenic microRNAs Anti-fibrotic factors	↑ Angiogenesis ↑ EC survival ↑ Vascular density ↓ Inflammation	Multiple growth factors Anti-apoptotic signaling Inflammatory cytokines ↓	[87]

Symbols: ↑, increased; ↓, decreased.

**Table 4:** EV-Mediated SMC Phenotype Modulation.

EV Source	Key Cargo	Contractile Markers	Proliferation/Migration	Phenotype Effect	Key Studies
Healthy EC-EVs (LSS)	miR-143/145 (30-fold enriched with KLF2)	↑ α-SMA ↑ SM22α ↑ SM-MHC ↑ Calponin ↑ Smoothelin	↓ Proliferation ↓ Migration ↓ Dedifferentiation	Maintain/promote contractile phenotype	[76,97]
EC-EVs (impaired flow/atherogenic)	Inflammatory mediators, pro-proliferative factors	↓ α-SMA ↓ SM22α ↑ Vimentin ↑ Osteopontin	↑ Proliferation ↑ Migration ↑ Synthetic phenotype	Promote synthetic/proliferative phenotype	[37]
Adipose-derived MSC-EVs	miR-125b, miR-146a, anti-inflammatory proteins	Preserved	↓↓ Proliferation ↓↓ Migration ↓ Intimal hyperplasia	Inhibits synthetic phenotype, reduces neointima	[93]
Bone marrow MSC-EVs	miR-125b (targets Myo1e)	Preserved	↓ Proliferation ↓ Migration ↓ Invasion	Suppresses SMC proliferation/migration	[95]
MSC-EVs with AT2R overexpression	AT2R pathway activation, dual-action cargo	Preserved/enhanced	↓ Proliferation ↓ Migration (while supporting EC)	Double benefit: ↓ SMC + ↑ EC	[86]
Platelet-EVs	Growth factors (PDGF), miR-223	Variable (context dependent)	↑ Proliferation (acute phase) Variable (context dependent)	Context dependent: can promote or inhibit	[85]
Small EVs + Collagen VI	Surface collagen VI (tetraspanin-bound)	None	↑ Migration (via focal adhesions) ↑ Invasion (β1 integrin/FAK/Src, Arp2/3)	Enables directed SMC migration	[96]

Symbols: ↑, increased; ↓, decreased.

**Table 5:** EVs in Immunomodulation and Macrophage Polarization.

EV Source	Immunomodulatory Cargo	Macrophage Effects	Inflammatory Markers	Functional Outcomes	Key Studies
MSC-EVs (general)	miR-146a, miR-125b IL-10, TGF-β Anti-inflammatory proteins Immunomodulatory factors	M1 → M2 polarization ↑ Arg1, CD206 ↓ iNOS, CD86, CD11b ↓	TNF-α ↓ IL-1β, IL-6 ↓ IL-12 ↑ IL-10 ↑ TGF-β ↓	↓ Inflammation, ↑ Tissue repair, ↑ Wound healing, ↓ Fibrosis	[100,101]
Cardiosphere-derived Cell-EVs	Cardioprotective factors Anti-inflammatory cargo M2-promoting signals	M2 polarization (24 h) ↑ Arg1/iNOS ratio ↑ CD163+ monocytes	↓ Pro-inflammatory cytokines ↑ Anti-inflammatory mediators	↑ Cardiac repair ↓ Adverse remodeling ↑ Function recovery	[87]
M2 Macrophage-EVs	LncRNA AK083884 Anti-inflammatory factors Mitochondria IL-10, TGF-β promote	M2 polarization (positive feedback) Inhibits glycolysis	↓ Pro-inflammatory ↑ Anti-inflammatory ↑ SOCS2/STAT6	↑ Sustained M2 state, ↑ Resolution, Transfers mitochondria to cardiomyocytes	[104]
M1 Macrophage-EVs	Proinflammatory mediators But context-dependent effects	Context-dependent: May paradoxically promote M2 polarization	Variable dependent on microenvironment-	Complex; depends on cell state and reception of local signals	[40,99]
HA-modified LSS-EVs (HA@LSS-EVs)	miR-34c-5p (enriched) HA targeting (CD44^+^) TGIF2-targeted	M1 → M2 repolarization in plaques Targeted to CD44^+^- macrophages	↓ Plaque inflammation ↑ TGF-β-Smad3 pathway ↓ Proinflammatory	↓ Atherosclerotic plaque progression, ↑ Plaque stability	[8]
M2-EVs fused to platelet membranes	M2 cargo Platelet targeting (P-selectin, GPIIb/IIIa) Hybrid vesicle	Targeted delivery to atherosclerotic plaques Enhanced M2 effects	↓ Plaque inflammation ↑ Targeted anti-inflammatory	↑ Plaque targeting ↑ Therapeutic efficacy ↓ Off-target effects	[109]
Platelet-EVs (thrombotic context)	Tissue factor (TF) Also: miR-223, miR-320b Dual pro/anticoagulant	Modulates inflammatory response at injury sites	↑ TF-dependent coagulation ↓ MAPK/NF-κB (miRNA-mediated)	Dual role: ↑ hemostasis, but ↓ excessive inflammation	[79]

Symbols: ↑, increased; ↓, decreased; →, transition.

**Table 6:** EV-Eluting Stent Systems—Proof-of-Concept Studies.

Study	[17]	[20]	[57]
Design/Model	Rat model: Renal I/R injury Abdominal aortic stenting 28-day follow-up	Rat model: Mal-efferocytosis Stenting Neointimal formation assessment	In vitro and ex vivo: EK/SMC co-culture Porcine coronary arteries
EV Source	MSC-derived exosomes (bone marrow MSCs)	MSC-derived exosomes	rhCollagen III-based ECM-mimetic coating (biomimetic approach)
Release Mechanism	ROS-sensitive polymer matrix: Thioketal linkages Inflammatory trigger Controlled release	Lp-PLA2-sensitive: Liposome-based chambers Enzyme-triggered release Adaptive to disease activity	ECM-mimicking dual bionics: Nanofiber structure Bioactive collagen III Passive release
Measured Results	Reendothelialization rate Neointimal hyperplasia Kidney function Muscle regeneration	Efferocytosis (clearance of apoptotic cells) Neointimal thickness Foam cell formation	EC proliferation SMC phenotype Anticoagulation Anti-inflammatory response
Key Findings	↑ Accelerated EC coverage and DES ↓ Neointima and BMS Systemic effects: ↑ kidney protection, ↑ hindlimb regeneration	↑ Increased efferocytosis ↓ Neointimal thickness increased by 42% and BMS (*p* < 0.05) ↓ Foam cell formation via CD36 regulation	↑ EC proliferation ↑ Contractile SMC phenotype ↓ Coagulation ↓ Inflammation
Significance	First demonstration of a ROS-sensitive exosome-releasing stent with both local and systemic regenerative effects	Demonstrated enzyme-sensitive release with spatial specificity; exosomes regulate efferocytosis via the SLC2a1/STAT3/RAC1/CD300a pathways	ECM-mimetic approach recapitulates the native vascular microenvironment; rhCollagen III provides anticoagulant, anti-inflammatory, and healing-promoting effects

Symbols: ↑, increased; ↓, decreased.

**Table 7:** Key scientific and Translational Priorities for EV-Inspired Cardiovascular Devices.

Challenge Category	Specific Issues	Impact	Proposed Solutions	Current Situation
Manufacturing and Scalability	Low EV yields (current methods) Batch-to-batch variability Single stent requires 10^11^–10^12^ EVs Upstream cell culture variability	Not enough EVs can be produced for clinical use; Cost prohibitive; Quality inconsistency	Bioreactor-based manufacturing Automated systems Tangential flow filtration Chromatography purification Standardized cell culture protocols	Preclinical phase; Few companies developing scale-up production; Still needs validation
EV Heterogeneity	Multiple subpopulations Size/density variations Cargo composition differences Uncertain which subpopulation is therapeutic	“Active pharmaceutical ingredient” definition is difficult; Potency variability; Regulatory challenges	Single EV analysis technologies Subpopulation isolation methods Multi-omics characterization Defining critical quality attributes (CQAs)	Emerging technologies; ISEV MISEV2023 provides guidelines; CQAs being defined
Formulation and Storage	Freeze–thaw sensitivity Organic solvent sensitivity Instability at room temperature Aggregation during lyophilization Storage at −80 °C is impractical	Standard coating techniques cannot be used; Clinical distribution challenges; Shelf-life limitations	Lyophilization with cryoprotectants Trehalose/sucrose stabilization Polymer encapsulation Cold chain optimization Biomimetic synthetic alternatives	Optimization ongoing; Various formulations in development; CDNs/PFLs show promise
Regulatory Framework	Unclear classification (drug, biologic, or device) No established guidelines Fragmented oversight (US: CBER/CDER) Impact analysis challenges	Unclear approval process; Expensive/lengthy trials; Difficulty comparing products	Early engagement with regulatory agencies Developing consensus CQAs Generating impact analyses Harmonizing international standards Following ISEV guidelines	FDA/EMA beginning to address; ISEV MISEV2023 provides research standards; Clinical trials emerging
Quality Control and Characterization	No standardized quality control methods Multiple measurement techniques yield different results Difficult to define efficacy Lack of reference standards	Cannot guarantee product consistency; Difficult to compare studies; Regulatory approval challenges	Standardized quality control panels Reference materials Validated efficacy assays Multimethod orthogonal characterization GMP manufacturing protocols	ISEV working on standards; Some commercial quality control services available; Still evolving
Clinical Trial Design	Nonlinear dose–response Timing of intervention is critical Patient heterogeneity Multiple mechanisms of action Traditional endpoints may miss benefits	Standard trial designs may fail; Dose selection is difficult; Patient stratification is unclear	Adaptive trial designs Multiple dose arms Patient stratification (diabetes, kidney function) New endpoints (OCT, endothelial function) Biomarker-guided approaches	Early-phase trials planned; Dose-finding studies needed; Endpoint validation ongoing
Mechanical Gaps	Cell type specificity mechanisms unclear Relative contribution of cargo components unknown Immunogenicity of allogeneic EVs uncertain Long-term effects not studied	Cannot be rationally optimized; Unanticipated effects possible; Safety concerns	Basic research investment Single-EV multi-omics Mechanistic preclinical studies Immunogenicity assessment Long-term safety studies	Active area of research; Many mechanistic studies ongoing; Lack of long-term data
Cost and Reimbursement	High manufacturing costs (current methods) Unclear pricing strategy Unclear reimbursement pathway Lack of cost-effectiveness data	Market access challenges; Difficult healthcare system adoption	Scaling to reduce costs Value-based pricing models Health economic studies Demonstrate long-term benefit compared to DES Reduced complications = cost savings	Economic analyses needed; Pricing strategy under development; Comparative effectiveness studies needed

## Data Availability

No new data were created or analyzed in this study. Data sharing is not applicable to this article.

## Abbreviations

EV	extracellular vesicle(s)
sEV	small extracellular vesicle(s)
lEV	large extracellular vesicle(s)
DES	drug-eluting stent(s)
DCB	drug-coated balloon(s)
SMC	smooth muscle cell(s)
ECM	extracellular matrix
MVB	multi-vesicular body
ILV	intraluminal vesicle
ESCRT	endosomal sorting complex required for transport
RBP	RNA-binding protein(s)
OCT	optical coherence tomography
EPC	endothelial progenitor cell(s)
